# Technical Considerations for Robotic Pediatric Lobectomy

**DOI:** 10.1016/j.atssr.2026.03.003

**Published:** 2026-03-24

**Authors:** Vikram Krishna, Claire Perez, Drew Bolster, Kellie Knabe, Raffaele Rocco, Philicia Moonsamy, Eugene S. Kim, Harmik J. Soukiasian, Andrew R. Brownlee

**Affiliations:** 1 Division of Thoracic Surgery, Cedars-Sinai Medical Center, Los Angeles, California; 2 Division of Pediatric Surgery, Cedars-Sinai Medical Center, Los Angeles, California

## Abstract

Robotic thoracic surgery in pediatric patients is technically challenging due to narrow anterior-posterior and lateral chest dimensions, which increases instrument collisions. Consequently, lung tumors in children are traditionally resected through a thoracotomy. We present a reproducible approach to robotic pediatric lobectomy in a 12-year-old girl with a biopsy specimen-proven left lower lobe mucoepidermoid carcinoma. Our technique uses 4 robotic ports, with 3 posterior ports in the ninth intercostal space and an anterior stapling port positioned 1 to 2 interspaces higher, achieving 6 to 8 cm of spacing. Preoperative marking and careful patient selection enable a feasible robotic lobectomy within the constrained pediatric thorax.

Robotic-assisted thoracoscopic surgery has gained increasing acceptance in the surgical management of lung cancer, largely due to improved perioperative outcomes when compared with open thoracotomy.^1^ Studies demonstrate that robotic approaches are associated with lower rates of conversion to open procedures compared with video-assisted thoracoscopic surgery, with reduced postoperative pain, shorter lengths of stay, and improved clinical recovery.^2^

Despite these advantages, pediatric lung cancer has historically been managed through thoracotomy, and use of robotic platforms in children remains uncommon because of cost considerations, lack of formal training, and limitations imposed by smaller thoracic dimensions and instrument size.^3^ However, early reports in pediatric populations suggest that robotic techniques may enhance operative precision and dissection efficiency while decreasing technical burden.^4^ In this report, we describe a case of robotic-assisted lobectomy in a pediatric patient, highlighting technical considerations required for the pediatric thorax.

A 12-year-old girl was evaluated for a chronic cough and found on computed tomography to have a well-defined lesion located in the superior segment of the left lower lobe (Figure 1). Tissue diagnosis was obtained by way of a shape-sensing robotic-assisted bronchoscopy, which demonstrated mucoepidermoid carcinoma involving the bronchial wall. As a result of these findings, surgical resection was pursued using a robotic-assisted approach. Final histopathologic analysis confirmed a T2 N0 mucoepidermoid carcinoma.

**Figure 1:**
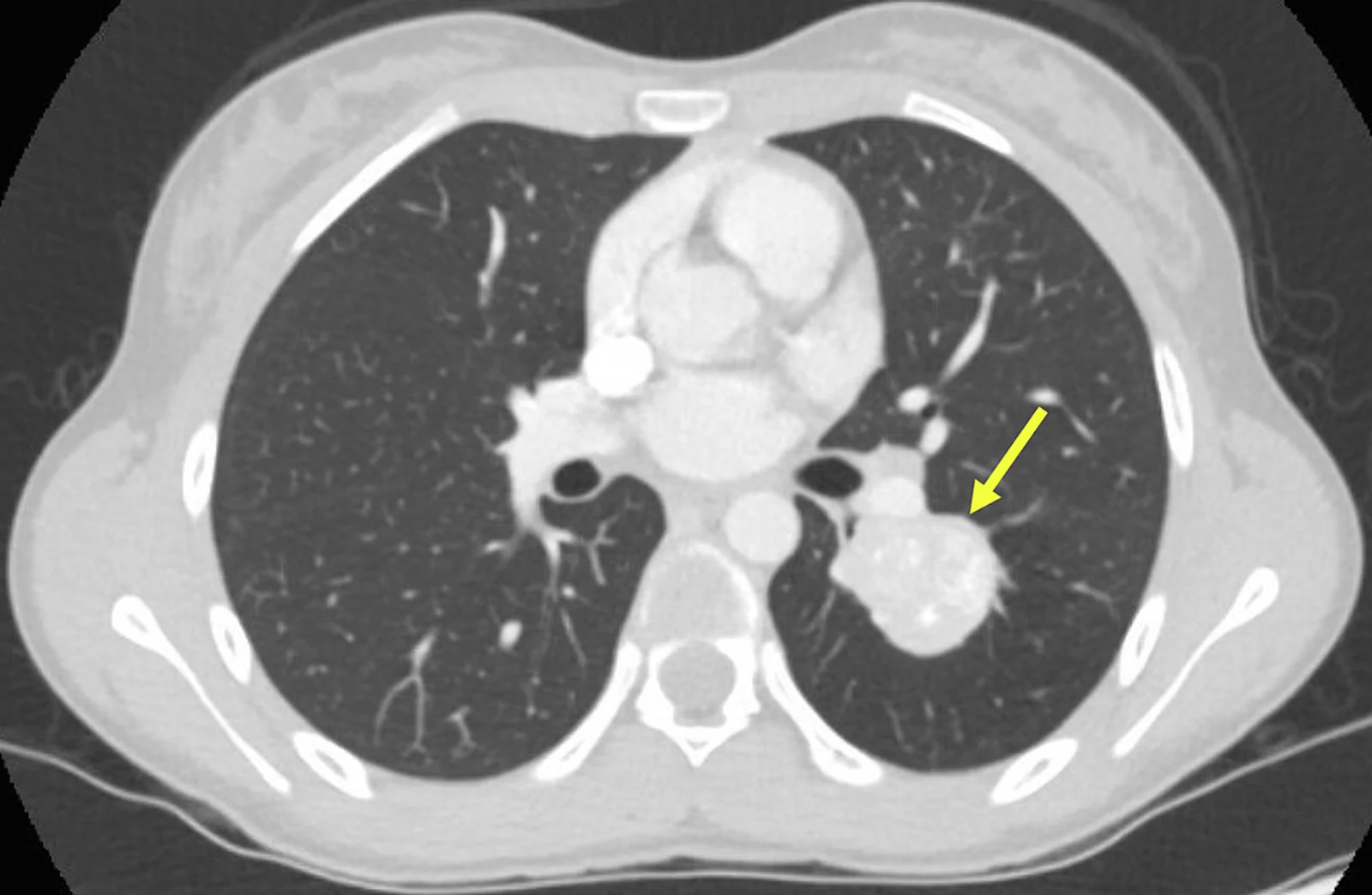
Computed tomography of the chest showed a well-circumscribed mass located in the superior segment of the left lower lobe (arrow).

Institutional review board approval was not required. The patient’s parents consented to deidentified pictures/videos collected during this procedure. Cedars-Sinai Medical Center is a teaching hospital, and this practice is standard and included in procedure consent, which was obtained before surgery.

Patient selection is critical, and all cases are evaluated within a multidisciplinary team, including pediatric oncology, pediatric surgery, and thoracic surgery, with particular attention to chest size, anatomy, tumor characteristics, and biology. The pediatric thorax presents unique spatial constraints due to its reduced anterior-posterior and lateral dimensions, which can limit port distance and increase the risk of robotic arm collision. Our patient was 154.9 cm and 42.2 kg. To address this, port sites are carefully marked preoperatively to optimize spacing and minimize external and internal interference.

Our configuration uses 4 robotic ports, with 3 positioned posteriorly along the ninth intercostal space and an anterior stapling port placed 1 to 2 interspaces superiorly (Figure 2). This arrangement maintains ∼6 to 8 cm between ports and closely mirrors our adult robotic lobectomy setup, despite the narrower pediatric chest. The da Vinci Xi robotic platform (Intuitive Surgical) is docked in a standard fashion. The more compliant pediatric mediastinum requires lower insufflation pressures (generally 6-8 mm Hg), which are adjusted based on exposure and physiologic response.

**Figure 2:**
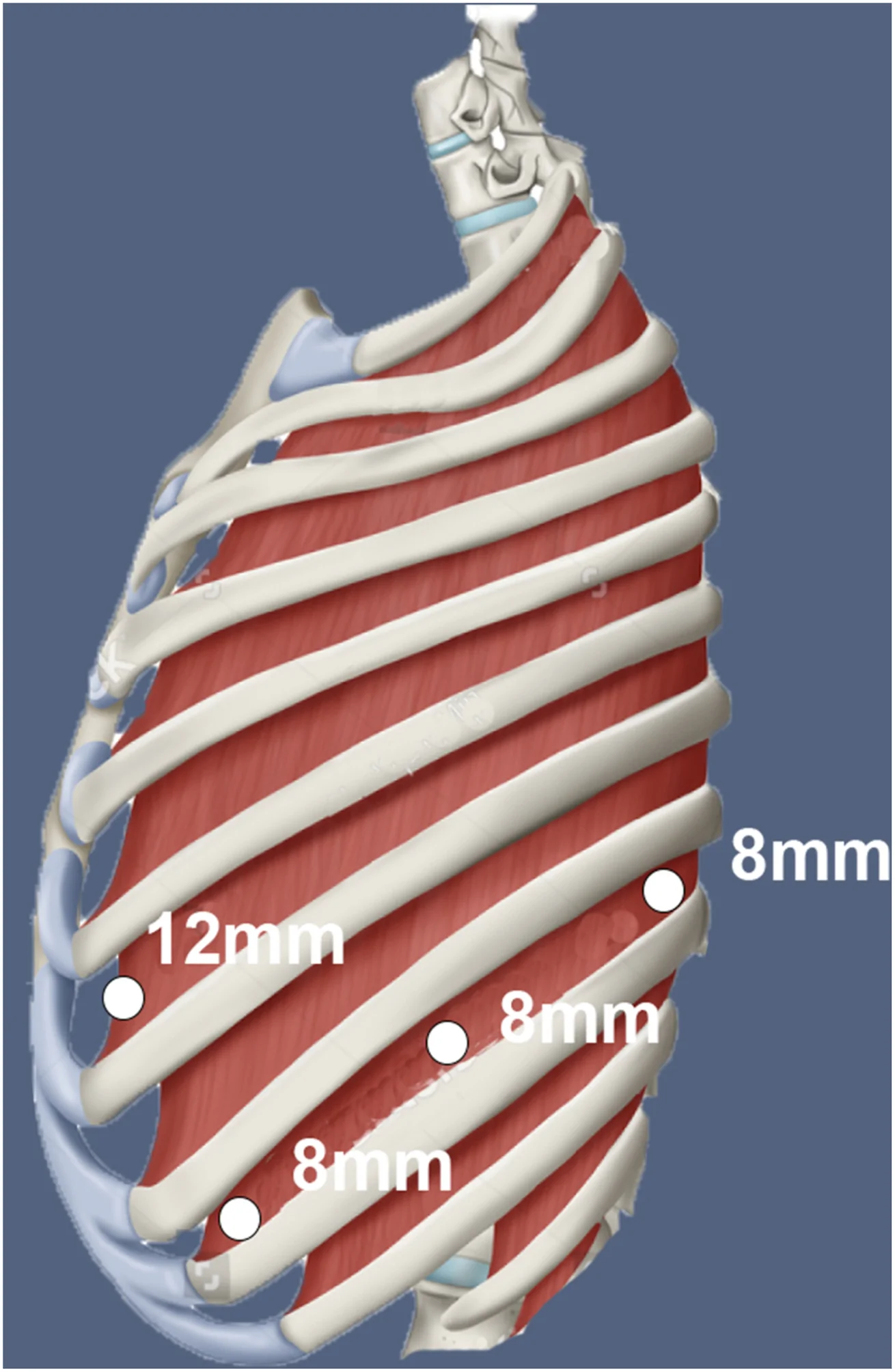
Our standard approach uses 3 posterior robotic ports placed in the ninth intercostal space, with the anterior stapling port positioned 1 to 2 interspaces higher.

The operation proceeds with posterior mediastinal dissection and systematic lymphadenectomy to fully expose the relevant hilar and mediastinal structures. The lung is initially retracted anteriorly and apically using a cigar sponge to expose the inferior pulmonary ligament, which is divided with bipolar energy (Supplemental Video). This maneuver allows clear appreciation of the tumor’s size and its relationship to adjacent structures (Figure 3), particularly in cases where the lesion encroaches on neighboring lobes. Dissection is directed toward the plane of the pulmonary artery, which is notably thinner in pediatric patients and therefore requires meticulous handling. Despite this, vascular control is achieved using techniques analogous to those used in adults.

**Figure 3:**
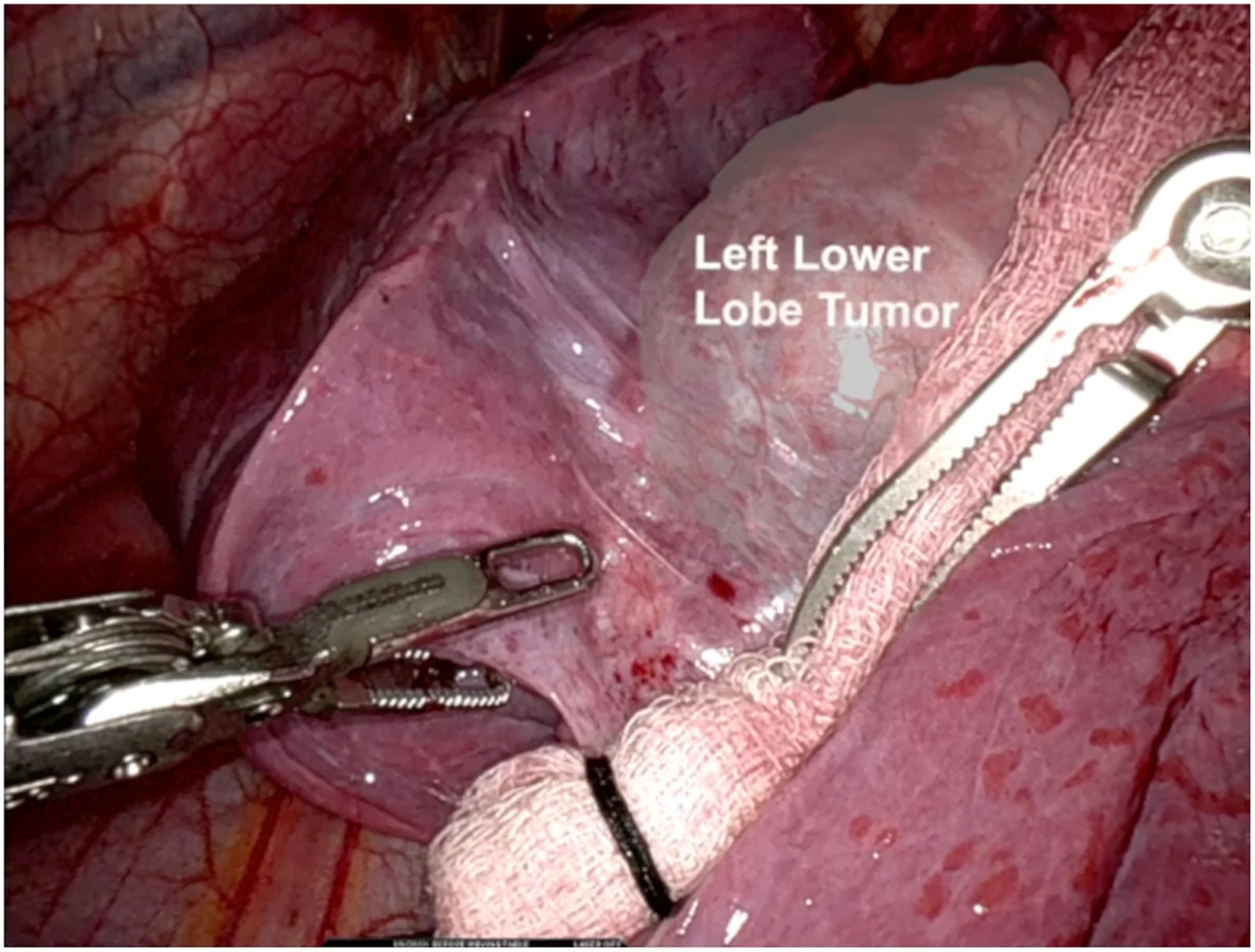
After the lung is retracted anteriorly and apically, a large tumor in the left lower lobe can be appreciated as it encroaches on the upper lobe.

As dissection progresses, the lung is retracted posteriorly to expose the fissure. The fissure is developed along the avascular plane, following the plane of Leriche, with the goal of fully exposing the pulmonary artery and connecting the anterior and posterior dissections. Bipolar cautery is avoided in the fissure, whenever possible, to reduce the risk of postoperative air leak. Instead, vessel loops are used to elevate the parenchyma and create a controlled tunnel, allowing safe stapled division, rather than sharp or energy-based dissection. This technique is particularly important in the setting of large tumors, where careful isolation of the vessel and elevation of surrounding tissue away from both the pulmonary artery and tumor are essential for safe stapler passage.

Stapling is performed using a combination of 8-mm and 12-mm robotic staplers. In smaller patients, the 8-mm stapler can be used exclusively, including for fissure division, permitting a smaller anterior port size while maintaining adequate control. Special attention is paid to creating sufficiently large windows around vascular structures before stapler introduction, because these windows may appear smaller once a 12-mm stapler is advanced into the chest. A vessel loop is often used to encircle and elevate the pulmonary artery, facilitating additional posterior dissection and ensuring a safe margin for transection. Once an adequate window is established inferior to the artery, a white vascular load is used for division. The pulmonary vein and left lower lobe bronchus are divided in a similar fashion.

The procedure concludes with excision of lymph node stations 5 and 6 in accordance with oncologic principles, followed by specimen retrieval and administration of intercostal nerve blocks.

## Comment

Mucoepidermoid carcinoma of the lung is an uncommon malignancy in children and is frequently misdiagnosed as more common conditions such as pneumonia or asthma.^5^ Despite its rarity, the disease is typically characterized by indolent behavior and an excellent prognosis, particularly in patients with stage I or II disease. After complete surgical resection, long-term outcomes are favorable, with low rates of local recurrence and distant metastasis.

This case demonstrates the feasibility of robotic-assisted lobectomy in the pediatric population when anatomic constraints are deliberately addressed. Successful pediatric robotic lobectomy depends on careful patient selection after multidisciplinary discussion with the pediatric team and family, and feasibility is determined by patient size, chest shape, and age. Preoperative port planning to achieve 6 to 8 cm of clearance, along with judicious use of insufflation in the more compliant pediatric chest is essential to minimize instrument collision and maintain physiological safety. Strategic elevation of the anterior port by 1 to 2 intercostal spaces relative to the posterior ports increases the working domain within the pediatric thorax and permits feasible deployment of longer and larger robotic instruments, including staplers. With thoughtful adherence to pediatric-specific technical principles and anticipating spatial limitations of the constrained chest, robotic lobectomy can be effectively performed in the pediatric population.
